# Is Genetic Background Important in Lung Cancer Survival?

**DOI:** 10.1371/journal.pone.0005588

**Published:** 2009-05-29

**Authors:** Linda S. Lindström, Per Hall, Mikael Hartman, Fredrik Wiklund, Kamila Czene

**Affiliations:** 1 Department of Medical Epidemiology and Biostatistics, Karolinska Institute, Stockholm, Sweden; 2 Department of Surgery, Karolinska University Hospital, Stockholm, Sweden; Ohio State University Medical Center, United States of America

## Abstract

**Background:**

In lung cancer, a patient's survival is poor with a wide variation in survival within the stage of disease. The aim of this study was to investigate the familial concordance in lung cancer survival by means of analyses of pairs with different degrees of familial relationships.

**Methods:**

Our population-based Swedish family database included three million families and over 58 100 lung cancer patients. We modelled the proband (parent, sibling, spouse) survival utilizing a multivariate proportional hazard (Cox) model adjusting for possible confounders of survival. Subsequently, the survival in proband's relative (child, sibling, spouse) was analysed with a Cox model.

**Findings:**

By use of Cox modelling with 5 years follow-up, we noted a decreased hazard ratio for death in children with good parental survival (Hazard Ratio [HR] = 0.71, 95% CI = 0.51 to 0.99), compared to those with poor parental survival. Also for siblings, a very strong protective effect was seen (HR = 0.14, 95% CI = 0.030 to 0.65). Finally, in spouses no correlation in survival was found.

**Interpretation:**

Our findings suggest that genetic factors are important in lung cancer survival. In a clinical setting, information on prognosis in a relative may be vital in foreseeing the survival in an individual newly diagnosed with lung cancer. Future molecular studies enhancing the understanding of the underlying mechanisms and pathways are needed.

## Introduction

Lung cancer remains the leading cause of death in the Western world and in spite of adequate surgery, adjuvant chemotherapy and radiotherapy, outcome remains poor with 5-year overall survival rates of less than 20% [1]–[3]. Important factors influencing lung cancer survival will include metastatic potential of the tumour, response to treatment, behavioural and sociodemographic characteristics [1].

The genetic background of a patient with lung cancer might be essential for the ability of the tumour to metastasis, since generally the mechanism by which cells try to colonise at distant sites is surprisingly inefficient [4]–[7]. Recently, several molecular studies have found genetic factors associated with lung cancer survival [8]–[12], and previously published data from our group on familial survival concordance in parents and children with lung cancer does not distinguish but implies the importance of both genetic and environmental factors [13]. Smoking is truly the main environmental risk factor for lung cancer, nonetheless the overall effect of smoking on lung cancer survival has been seen to at most, in certain histologies and in women, increase the risk of dying with around 30% [14]–[16].

As a first nation-wide Swedish population-based epidemiological study, we analysed the familial correlation in lung cancer survival. Our aim was to disentangle the importance of genetic and environmental factors in lung cancer survival by analysing family pairs with different degree of relationship.

## Methods

### Swedish population-based family data

Linkage of different records of personal information is possible in Sweden as each resident has a unique national registration number. Our study is based on a record linkage between several population-based registers; the Multi-Generation Register, the Swedish Cancer Register, the Cause of Death Register, and the Migration Register. Finally, additional linkages were made to the Censuses of 1960, 1970, 1980 and 1990 that holds information on individual socioeconomic status.

The Multi-Generation Register includes individuals born in Sweden from January 1932 through December 2001 with their biological parents. The proportion of false paternity is not known in the study cohort, but has been estimated to account for less than 5% in other European countries with similar registers [17], [18].

Incident cancers in Sweden since 1958 have been reported to the Swedish Cancer Register using a four digit diagnostic code according to the 7^th^ revision of the International Classification of Diseases (ICD-7), together with information on histopathological type. In the 1970s, the completeness of cancer registration (with cytological or histological verification) was assessed to be around 95% and has been regarded to be close to 100% since the 1990s [19]. In our study, lung cancer was defined as cancers coded as ICD-7 162 and tumour histology was categorised into five groups; adenocarcinoma (histopathological type 096), squamous cell carcinoma (histopathological type 146), small cell carcinoma (histopathological type 186), large cell carcinoma (histopathological type 196) and other histology. Deaths caused by lung cancer (underlying cause of death) were collected from the Cause of Death Register, which has a reported accuracy of 96% from 1961 onwards [20]–[22]. Information on cause of death was ascertained from death certificates filled in by treating physicians.

In the Multi-Generation Register each child exists only once while parents are present as many times as they have children. An individual can be in the database both as offspring and parent and parents are those that admit to parenthood at birth, thus not only married individuals. Our database comprised over 11 million individuals organized into around three million families, including more than 58 100 lung cancer patients.

Because treatment and thus survival may differ geographically in Sweden, area of diagnosis was obtained from the Cancer Register and categorized into 6 health care regions as defined by the health care structure of Sweden. Socioeconomic status was given in the Censuses and was categorized into following groups; blue collar workers, white collar workers, self employed, farmers and others.

### Statistical analysis

From our database we selected all pairs of parent-child, siblings and spouses concordantly diagnosed with a first primary invasive lung cancer. The outcome of interest was cause-specific lung cancer death within 5-years because it is a clinically relevant measure. The person-time at risk started at the date of lung cancer diagnosis and continued until emigration, end of follow-up (December 31, 2001), or death, whichever came first. In our register-based study we have complete follow-up. Out of 439 parent-child pairs, 63 sibling pairs, and 525 spouse pairs, 60 parents and 60 children, six younger siblings and nine older siblings and 90 husbands and 62 wives were censored, respectively. The rest of the individuals were either followed until end of follow-up or died due to lung cancer.

We limited our follow-up back to 1961 since the Cause of Death Registry has a high reported accuracy of 96% from 1961 onwards. We restricted our offspring analysis to 1991 and onwards, because complete data for parents of children who died from 1991 are available in the Multi-Generation Register, whereas before this date the data are incomplete. Accordingly, both children and siblings in our analyses were diagnosed between January 1991 and December 2001, while for parents and spouses the follow-up was unrestricted (January 1961 to December 2001). We selected husbands as proband for consistency with sibling analyses (oldest sibling was defined as proband) since generally husbands are older than their wives.

The survival in the proband (parent, sibling, spouse) was modelled employing a multivariate proportional hazards (Cox) model adjusting for the calendar year of diagnosis and age at diagnosis. The residuals from this model were used to describe proband survival compared with the cumulative baseline hazard, adjusting for calendar year of diagnosis and age at diagnosis, resulting in residual values below, above, and around zero. We, subsequently, categorized proband survival by defining groups according to quartiles of survival, with the better than expected survival group as the best quartile of survival, the expected survival group as the middle two quartiles of survival, and the worse than expected survival group as the worst quartile of survival. For simplicity, we refer to these categories as good, expected and poor. Depending on the modelled survival in proband the survival in proband's relative (child, sibling, spouse) was analysed with a multivariate proportional hazard model adjusting for possible confounders on survival such as calendar year of diagnosis, age at diagnosis, socioeconomic factors, county of diagnosis, tumour histology and gender.

The proportional hazard assumption for the main exposure variable was assessed using Schoenfeld's test statistics [23]; no significant deviation was noted for the family pairs studied. A level of 5% statistical significance was used. All data preparation and analysis was done using the SAS Statistical package, version 9.1, whereas Stata was used to test the proportional hazards assumption.

## Results

The number of parent-child, sibling and spouse pairs diagnosed with lung cancer and number of lung cancer-specific deaths are presented in Table 1. Descriptive factors such as period and age at diagnosis, tumour histology, socioeconomic status and gender distributions in relative pairs with lung cancer are presented in Table 2.

**Table 1:** Characteristics of parent-child, sibling and spouse pairs with primary lung cancer diagnosed in Sweden.

Pairs of relatives	Pairs with lung cancer	Concordant alive	Concordant dead
**All lung cancer**			
Parent^a^-Child^b^	439	32	256
Sibling-Sibling^b^	63	13	30
Spouse-Spouse^a^	525	39	301
**Non-small cell lung cancer**			
Parent^a^-Child^b^	358	28	205
Sibling-Sibling^b^	49	12	18
Spouse-Spouse^a^	414	30	244

a Parents (Spouses) diagnosed between January 1961 and December 2001.

b Children (siblings) diagnosed between January 1991 and December 2001.

**Table 2:** Period, age, histology, socioeconomic status and gender distributions in relative pairs with lung cancer.

	Relative pairs					
	Parent - Child		Sibling - Sibling		Spouse – Spouse	
	Parent	Child	Older sibling	Younger sibling	Husband	Wife
**Period**						
1961–1969	59 (13%)				45 (9%)	31 (6%)
1970–1979	117 (27%)				103 (20%)	65 (12%)
1980–1989	155 (35%)				157 (30%)	175 (33%)
1991–2001	108 (25%)	439 (100%)	63 (100%)	63 (100%)	220 (42%)	254 (48%)
**Age**						
<50	7 (2%)	94 (21%)	12 (19%)	21 (33%)	23 (4%)	34 (6%)
50–59	58 (13%)	235 (54%)	29 (46%)	31 (49%)	103 (20%)	106 (20%)
60–69	148 (34%)	110 (25%)	22 (35%)	11 (17%)	193 (37%)	191 (36%)
70+	226 (51%)				206 (39%)	194 (37%)
Mean age	76	60	61	58	73	72
**Tumour histology**						
Adenocarcinoma	76 (17%)	167 (38%)	29 (46%)	22 (35%)	88 (17%)	133 (25%)
Squamous cell carcinoma	155 (35%)	75 (17%)	13 (21%)	11 (17%)	195 (37%)	123 (23%)
Small cell carcinoma	24 (6%)	61 (14%)	9 (14%)	9 (14%)	45 (9%)	75 (14%)
Large cell carcinoma	143 (33%)	106 (24%)	9 (14%)	16 (25%)	149 (28%)	141 (27%)
Other	41 (9%)	30 (7%)	3 (5%)	5 (8%)	48 (9%)	53 (10%)
**Socioeconomic status (SES)**						
Blue collar workers	242 (55%)	183 (42%)	28 (44%)	35 (56%)	284 (54%)	253 (48%)
White collar workers	95 (22%)	172 (39%)	24 (38%)	17 (27%)	137 (26%)	180 (34%)
Self employed	37 (8%)	25 (6%)	2 (3%)	3 (5%)	55 (10%)	44 (8%)
Farmers	24 (5%)	1 (0%)	0 (0%)	1 (2%)	11 (2%)	12 (2%)
Others	41 (9%)	58 (13%)	9 (14%)	7 (11%)	38 (7%)	36 (7%)
**Gender**						
Female	112 (26%)	219 (50%)	38 (60%)	37 (59%)		525 (100%)
Male	327 (74%)	220 (50%)	25 (40%)	26 (41%)	525 (100%)	

The hazard ratio in the proband's relative (child, sibling, spouse) depending on proband survival, was estimated by use of a multivariate (Cox) model, see Table 3. In the parent-child analyses (adjusting for calendar year of diagnosis, age at diagnosis, socioeconomic factors, county of diagnosis, tumour histology and gender) children with good parental survival had a decreased hazard ratio for death of 0.71 (95% CI = 0.51 to 0.99), compared to children with poor parental survival. Also, in siblings, with good proband survival, the hazard ratio for death was significant at 0.14 (95% CI = 0.030 to 0.65). Finally, in spouses, no significant effect on spouse survival was seen. Choosing the proband spouse to be husband or wife had no impact on the results (results not shown).

**Table 3:** Risk of lung cancer-specific death in proband's relative (child, sibling, spouse) depending on proband survival.

Pairs of relatives	Survival in proband^a^	Number of relatives to proband	Number of deaths in relative to proband	Risk of lung cancer related death in proband's relative			
				Adjusted^a^	Trend test	Adjusted^c^	Trend test
				HR (95% CI)	p values^b^	HR (95% CI)	p values^b^
Parent^d^-Child^e^					0.05		0.04
	Good	109	67	0.73 (0.52–1.00)		0.71 (0.51–0.99)	
	Expected	221	157	0.87 (0.68–1.16)		0.86 (0.65–1.13)	
	Poor	109	83	1.0 ref.		1.0 ref.	
Sibling-Sibling^e^					0.02		0.05
	Good	15	4	0.24 (0.072–0.81)		0.14 (0.030–0.65)	
	Expected	32	25	1.42 (0.66–3.05)		1.26 (0.48–3.27)	
	Poor	16	10	1.0 ref.		1.0 ref.	
Spouse-Spouse^d^					0.32		0.26
	Good	131	97	0.87 (0.66–1.15)		0.85 (0.64–1.13)	
	Expected	262	196	0.91 (0.71–1.15)		0.90 (0.70–1.15)	
	Poor	132	102	1.0 ref.		1.0 ref.	

a Multivariate proportional hazard (Cox) model adjusted for calendar year of diagnosis and age at diagnosis.

b One degree of freedom.

c Multivariate proportional hazard (Cox) model adjusted for calendar year, age and place of diagnosis, socioeconomic status, tumour histology and gender.

d Parents (Spouses) diagnosed between January 1961 and December 2001.

e Children (siblings) diagnosed between January 1991 and December 2001.

Adjusting the proband multivariate proportional hazards (Cox) model for all covariates such as calendar year of diagnosis, age at diagnosis, socioeconomic factors, county of diagnosis, tumour histology and gender, resulted in very similar estimates but with somewhat wider confidence intervals. Children, siblings and spouses with good proband survival had a decreased hazard ratio for death of 0.743 (95% CI = 0.532 to 1.039), 0.183 (95% CI = 0.045 to 0.736) and 0.87 (95% CI = 0.651 to 1.150), respectively.

We performed additional sub-analyses only including non-small cell lung cancer. In the non-small cell lung cancer analyses children, siblings and spouses with good proband survival had a decreased hazard ratio for death of 0.69 (95% CI = 0.48 to 0.99), 0.13 (95% CI = 0.019 to 0.94) and 0.78 (95% CI = 0.56 to 1.07) respectively, compared to children, siblings and spouses with poor proband survival. In none of these analyses a significant trend for the categories good, median and poor survival was achieved. We had no power to separately analyse small cell lung cancer.

## Discussion

In this Swedish population-based study, we show that lung cancer survival in an individual is dependent on the lung cancer survival in his/her parent or sibling. However, no survival correlation was seen in spouses. Our large population-based study has several strengths, including an almost complete ascertainment of cancers along with a complete follow-up of lung cancer patients.

Our estimates were robust showing only small differences when contrasting the fully adjusted model, which included calendar year of diagnosis, age at diagnosis, socioeconomic factors, county of diagnosis, tumour histology and gender, with the unadjusted model (only adjusted for calendar year of diagnosis and age at diagnosis). Since Swedish national registries are highly complete and accurate [19]–[22] and Swedish lung cancer survival increase has been modest [24], we believe that our retrospective cohort study of cancer prognosis will be almost as accurate as had we performed the same study in a prospective setting.

A limitation of our study was the absence of information on stage of disease as well as treatment because such information is not included in the Swedish Cancer Register and therefore not in our database. However, since the routinely used prognosticators for lung cancer poorly describe lung cancer outcome compared to many other malignancies this may not have effected the results or their interpretation. In addition, we argue that adjusting for such covariates in the analysis is inappropriate. If familial clustering of prognosis reflects a genuine biologic phenomenon, it should be mirrored in established prognostic factors and adjusting for them would consequently eliminate the association. Nevertheless, such information may have allowed a deeper understanding of the biologic mechanisms by which prognostic outcome is determined by cancer survival among probands.

Another potential limitation in the present study is the absence of information about smoking. However, we argue that our inability to adjust for smoking habits will have only minor effects on our results for several reasons. Firstly, the impact of smoking on lung cancer survival seems to be dependent on a number of factors such as sex, histological type and years since smoking cessation [14]–[16], [25] and the overall effect of smoking on lung cancer survival has been seen to at most, in certain histologies and in women, increase the risk with around 30% of dying [14]–[16]. Secondly, even if smoking is a truly established risk factor for lung cancer initiation, with strongly elevated disease risk observed among smokers, familial cases of lung cancer can not be attributed to shared smoking habits [26]. Finally, previous reports do not support higher correlation of smoking habits between siblings or parent-offspring's as compared to spouses [27]–[32].

Genetic variation has been associated with lung cancer survival. Recently, fifteen SNPs in the DNA repair pathway were associated with a significantly greater overall survival [33], and five gene signatures closely associated with relapse-free and overall survival among lung cancer patients were unravelled [34]. Also, the EGFR gene polymorphic simple sequence repeat (SSR) length [11], the Y/X polymorphism of the innate-immunity gene MBL2 with haplotypes [10], and glutathione-related genes have been associated with improved lung cancer survival [35].

The genetic background of an individual may influence the metastatic ability of a tumour. Moreover, allelic variants might modify the likelihood of tumour metastasis occurring through vital secondary events, such as deletions, amplifications, and epigenetic modulations in the metastatic cascade. Genetic variations may also affect the immune response, because small variations in the ability of an individual to mount an effective cytolytic defence, together with the tumour cell's ability to downregulate specific antigens, might be important in the metastatic cascade [36]. In addition, it is not unreasonable to hypothesize that response to therapy may be partly inherited. Interestingly, in a recent study, non-small-cell lung cancer (NSCLC) patients homologous for the ERCC1 118 (excision repair cross-complementing 1) exhibited a significantly better survival [37].

In conclusion, analyses of pairs with different familial relationships enable the distinction of genetic and environmental factors. Our findings suggest that genetic factors are important in lung cancer survival. Consequently, molecular understanding of the underlying mechanisms and pathways would help to better foresee the individual lung cancer prognosis.
